# Emission-related Heavy Metal Associated with Oxidative Stress in Children: Effect of Antioxidant Intake

**DOI:** 10.3390/ijerph17113920

**Published:** 2020-06-01

**Authors:** Brittany Killian, Tzu-Hsuen Yuan, Cheng-Hsien Tsai, Tina H. T. Chiu, Yi-Hsuan Chen, Chang-Chuan Chan

**Affiliations:** 1 Master of Public Health Degree Program, College of Public Health, National Taiwan University, No. 17, Xu-Zhou Road, Taipei 10055, Taiwan; bmariekillian@gmail.com; 2 Institute of Environmental and Occupational Health Science, College of Public Health, National Taiwan University, No. 17, Xu-Zhou Road, Taipei 10055, Taiwan; jamesfisher955@gmail.com (T.-H.Y.); u102014012@cmu.edu.tw (Y.-H.C.); 3 Innovation and Policy Center for Population Health and Sustainable Environment (Population Health Research Center, PHRC), College of Public Health, National Taiwan University, No. 17, Xu-Zhou Road, Taipei 10055, Taiwan; 4 National Taiwan University Hospital, Yunlin Branch, No.579, Sec. 2, Yunlin Rd., Douliu City, Yunlin County 64041, Taiwan; chtsa60@ntu.edu.tw; 5 Department of Nutritional Science, Fu-Jen Catholic University, No. 510, Zhongzheng Rd., Xinzhang Dist., New Taipei City 24205, Taiwan; 144601@mail.fju.edu.tw

**Keywords:** heavy metals, young children, oxidative stress, antioxidants, industrial pollution

## Abstract

Heavy metals, the common pollutants emitted from industrial activities, are believed to cause harmful effects, partially through the mechanism of elevated oxidative stress, and antioxidant intake has been hypothesized to provide a potential protective effect against oxidative stress. This study aims to investigate the heavy metal exposure and the associated oxidative damage of young children living near a petrochemical complex and to assess the protective effect of antioxidant intake. There were 168 children recruited from the kindergartens near a huge petrochemical complex, with 87 as the high exposure group and 81 as the low exposure group. Urinary concentrations of eleven metals were detected by inductively coupled plasma mass spectrometry, and four biomarkers of oxidative stress were measured in urine by liquid chromatography-tandem mass spectrometry. The food frequency questionnaire was collected to assess participants’ intake of antioxidants. Multiple linear regression was performed to determine the predictors of metals for oxidative stress and to measure the beneficial effect of antioxidants. Weighted quantile sum regression was performed to determine the contributors among metals to the oxidative stress. Results showed that high exposure group had significantly higher concentrations of chromium, manganese, nickel, arsenic, strontium, cadmium, and lead when compared to those in low exposure group. There was no obviously difference on the total antioxidant intake and dietary profile between two groups. The elevated levels of two oxidative stress markers were significantly associated with most of the urinary metal concentrations in all study subjects after adjusting confounders, while no significant association was found between oxidative stress and antioxidant intake. Among the metals, mercury and strontium showed the dominated contributions for elevated levels of oxidative stress. It concluded that higher metal exposure was associated with elevated oxidative stress but with no protective effect by antioxidant intake among the young children residents near a petrochemical industry.

## 1. Introduction

Environmental pollution, mainly from various industries and motor vehicles, was understood to be a health hazard for humans, with its negative effects spanning a wide range of diseases. Heavy metals are one source of environmental pollution that are of interest to researchers due to their potential for long-term negative health effects. One mechanism through which heavy metals cause damage to human health is oxidative stress. Many heavy metals have oxidation–reduction (redox) properties, which can contribute to the generation and overproduction of reactive oxygen species (ROS) when the antioxidant defenses are not sufficient to prevent this from occurring [1]. Consequently, the overproduction of ROS leads to oxidative damage. In recent years, many studies have found exposure to heavy metals to be associated with higher oxidative stress [2,3]. Industrial exposure to heavy metals has consistently been found to be associated with increased oxidative stress of the populations living in areas of high exposure [4,5,6]. Epidemiological studies have also linked proximity to industries to oxidative stress and related diseases [7,8,9]. 

High levels of oxidative stress during childhood may be a risk factor for various adult diseases. Studies have found that asthma, obesity, hypertension, severe disability, ADHD, and acute brain damage are associated with higher oxidative stress, and these diseases often begin developing during childhood [10,11,12,13,14,15]. Some studies have focused on even earlier stages of development, examining the effect of neonatal oxidative stress on later-life diseases [16,17]. Because many later life diseases have been linked to oxidative stress that occurred during early childhood, it is important to determine the causes of oxidative stress on young children and limit potential exposures. Young children are particularly susceptible to the effects of oxidative stress and environmental exposures due to their developing nervous, immune, digestive, respiratory, antioxidant, and reproductive systems [18]. During these developmental stages, harmful environmental exposures have potential to cause irreversible, life-long damage to cells [19]. Various observational studies have examined the ways heavy metal exposure affects young children. These studies have concluded that heavy metal exposure is a consistent predictor of urinary oxidative stress among children [20,21,22]. Other studies have reached similar conclusions after examining the effects of heavy metal exposure on infants and their mothers and adolescents [23,24].

In recent years, several experimental and observational studies have aimed to address the issue about the relationship between dietary antioxidant intake and oxidative stress. As children reach preschool age, their diets begin to more similarly resemble adult diets, allowing researchers to observe differences in dietary patterns among children. Studies have shown that for all ages of people, diets low in antioxidants have been linked to increased disease, especially when coupled with exposure to heavy metals [25]. Antioxidant status is of great importance because low antioxidant intake is often found to be associated with certain diseases. Previous studies examined young children’s dietary antioxidant intake and exposure to environmental chemicals, concluding that the relationship between antioxidant intake, environmental exposure to chemicals, and other physiological factors interact in a complex way [26]. Other studies have linked oxidative stress and dietary antioxidant intake to diseases, with many of them concluding that dietary supplementation of antioxidants may provide a beneficial effect on childhood developmental diseases such as asthma and neurological disorders [11,12,14,27,28].

Given the complex relationship between heavy metal exposure through industrial complexes, oxidative stress, and dietary antioxidant intake, the primary objective of this study was to investigate the heavy metal exposure and oxidative stress levels among young children living in the vicinity of a big petrochemical complex and to determine if heavy metal exposure is associated with oxidative stress among this population of young children. Another objective was to explore if dietary antioxidant intake provided a protective effect against oxidative stress related to environmental metal exposure. 

## 2. Materials and Methods 

### 2.1. Study Area 

The study area selected for this study was in Central Taiwan near a large petrochemical complex, which was built in 1998. There are 53 plants in the complex, including one thermal power plant with the capacity of 1.8 million kW of electricity, three oil refineries, two naphtha cracking plants, three cogeneration plants with the generation of 2.82 million kW of electricity, and other related plants. The production capacity of this complex has expanded to 540,000 barrels of oil per day and 2.9 million tons of ethylene per year [29].

Previous studies have concluded that various pollutants from the complex are a possible health risk for the local residents [30,31]. Among the petrochemical emission-related pollutants, the effect of toxic metal exposure was observed on residents and environment in the vicinity areas. For ambient air, the contents of many metals in PM_10_ samples were higher during the downwind season in the two townships nearby the complex [32], and the obviously increasing ambient concentrations of vanadium (V) were found in the closer areas of the complex [33]. For internal exposure biomarkers, urinary V and arsenic (As) levels displayed a concentration gradient in accordance with the distance-to-source gradient of V and as exposure [33], and the significant association between proximity to the petrochemical complex and heavy metals and oxidative stress biomarkers was found in teenagers and elderly population [7,34]. 

### 2.2. Study Subject

In this study, the study subjects selected were kindergarten children, ages 4–8, from four townships located in vicinity of the petrochemical complex. Initially, there were 104 children recruited from the two kindergartens in two townships closest to the petrochemical complex as the high exposure group, and there were 96 children recruited from the two kindergartens in two townships located farther from the petrochemical complex as the low exposure group. The sampling time period was conducted on January 10th and 11th, 2017. In addition, the urine sample and food frequency questionnaire were collected simultaneously from the subjects. Geographic information system (GIS) software (ArcGIS; Version 10.5; Environmental Systems Research Institute, Inc.) was used to determine the distances from the petrochemical complex to the study subject’s home address. A weighted average of the geographical exposure for each study subject was calculated using the number of hours the children spent at home and at school. 

**Figure 1:**
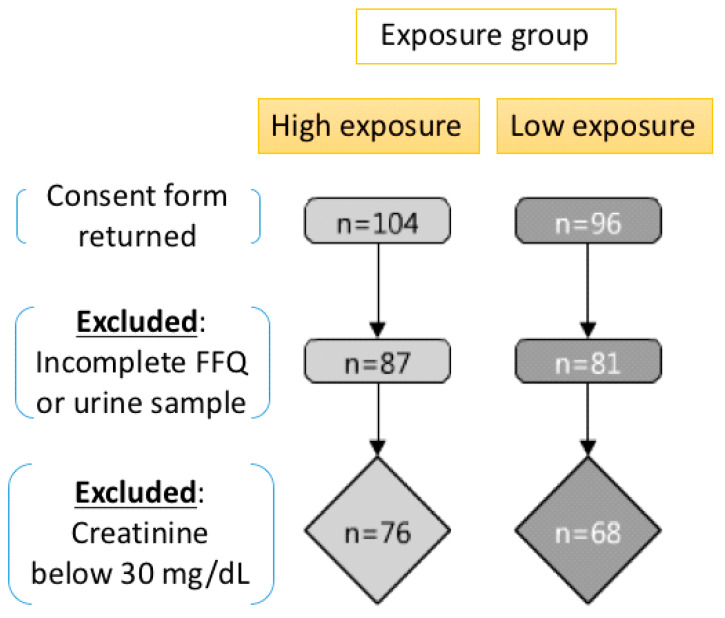
The study design and flow chart.

The informed consent of these 200 children were provided by the participants’ guardians, and the food frequency questionnaire (FFQ) investigation and urine sample collection were conducted at the four kindergartens. There were 32 participants excluded because of incomplete procedures, including returning both a morning urine sample and a completed food frequency questionnaire (FFQ), and therefore the total study subjects were 168 upon further analysis. Moreover, there were 24 samples with the urinary creatinine concentration below 30 mg/dL or above 300 mg/dL excluded from the study for urinary analysis in accordance with the World Health Organization (WHO) standards. The flowchart of this cross-sectional study was shown in Figure 1, and the Institutional Review Board (IRB) approval (201312017RIND) was obtained prior to initiation of the study.

### 2.3. Analysis of Exposure Biomarkers

A morning spot urine sample was collected by the guardians of the participants and then stored in a −20 °C freezer until analysis was performed. From the urine samples, levels of eleven heavy metals including vanadium (V), chromium (Cr), manganese (Mn), nickel (Ni), copper (Cu), arsenic (As), strontium (Sr), cadmium (Cd), mercury (Hg), thallium (Tl), and lead (Pb) were determined by inductively coupled plasma mass spectrometry (ICP-MS). To ensure accurate measurements, the urinary metal levels of standard reference materials (SERO, Billingstad, Norway) analyzed by our method were all within acceptable ranges provided by the standard reference materials. Additionally, the relative error of the ten spiked samples for each batch of the experiment was below 10% for these urinary chemicals. In addition, the measurement data was statistically analyzed when the recovery rate of each batch of the experiment higher than 85%. The one half of the method detection limit was used to represent the urinary metals level when the measurement was below the method detection limits. Urinary metal levels were adjusted using urinary creatinine concentrations, and these levels were log-transformed to fit a normal distribution for further statistical analysis.

### 2.4. Analysis of Oxidative Stress Biomarkers 

There were four biomarkers available to measure oxidative stress detected in our study, including four oxidative stress biomarkers (8-hydroxy-2′-deoxyguanosine (8-OHdG), 4-hydroxy-2-nonenal-mercapturic acid (4-HNE-MA), 8-isoprostaglandin F_2α_ (8-isoPF_2α_), and 8-nitroguanine (8-NO_2_Gua)). These four oxidative stress biomarkers were analyzed in urine samples with liquid chromatography-tandem mass spectrometry (LC-MS) using a previously-validated method that analyzed all four biomarkers at once [35]. Urinary oxidative stress biomarkers levels were adjusted using urinary creatinine concentrations, and these levels were log-transformed to fit a normal distribution for further statistical analysis.

### 2.5. Analysis of Antioxidant Intake 

Total antioxidant intake of the participants was determined using the FFQ, which was designed specifically for this study, as we could not find any FFQ in Taiwan that have been developed to measure antioxidant intakes and had been tested in children. FFQs are a tool used by nutritionists to determine food patterns and habits among study subjects. Moreover, it is a common method for calculating all nutrients from FFQ, including antioxidants. This method is commonly used in most studies of nutritional epidemiology that rely on FFQ to assess diet, such as the Swedish Mammography Cohort study [36]. Food items predictive of antioxidant nutrients or components of interests, such as vitamin C in children age 4 to 6, were identified by step-wise regression using dietary data collected in the 2005 Nutrition and Health Survey in Taiwan (NAHSIT). The more information of the regression was provided that vitamin C (antioxidant nutrient) was set as the dependent variable, while food items were set as independent variable in the regression formula. The stepwise regression then output foods that were most predictive of vitamin C intake [37]. Additional foods containing nonvitamin antioxidants that are commonly consumed by children were added to the FFQ, with consultation with a dietitian. The FFQ in this study included a list of foods and asked the participant how frequently they have consumed each food in the past month. Because young children cannot reliably provide their own dietary data, their parents or guardians served as representatives and completed the FFQs for them. 

To calculate total antioxidant intake per week per participant, each food in the questionnaire was matched to its corresponding antioxidant intake measured by the ferric-reducing ability of plasma (FRAP) value in mmol/100g using data from previous studies [38,39]. Most of the foods’ antioxidant content could be found through the previous two sources. Average portion sizes for children ages 4–8 were calculated using Taiwan’s National Health Survey data. Portion sizes were then multiplied by the antioxidant intake to get an approximate antioxidant intake per one-time consumption of each food. These numbers were then multiplied by the frequency with which the participant consumed a given food in a one-week period to find the total antioxidant intake per week. This FFQ included 67 specific food groups. 

### 2.6. Statistics

Basic characteristics and antioxidant intake between high and low exposures were compared using Student’s t-tests for continuous variables. For discrete categorical variables, the Chi-squared tests were performed. After adjusting for age, gender, household smoking, and parental work history at the petrochemical plant, differences on the levels of urinary metal and oxidative stress biomarkers between the high and low exposure participants were compared using analysis of covariance (ANCOVA). Multiple linear regression analysis was performed to determine the relationship between environmental exposure biomarkers, dietary intake, and oxidative stress. All linear regression models were with antioxidant intake and metal exposure biomarkers individually as independent variables and oxidative stress biomarkers individually as dependent variables adjusting for confounding factors. Weighted quantile sum (WQS) regression was performed to determine highest contributors among metal exposure biomarkers to each oxidative stress biomarker. A p-value of <0.05 was considered significant. All tests were performed using R Studio 3.2. WQS regression was performed using the gWQS package for R 3.5.1.

## 3. Results

### 3.1. Basic Characteristics 

Table 1 showed the basic characteristics of participants between the high and low exposure groups. Gender was significantly different between the two groups, with the high exposure and low exposure groups comprised of 57.41% males and 40.74% males, respectively. Age, household smoking, and percent of single parent households were not significantly different, but parental work history at the petrochemical plant was different between the two groups. Of the parents of high exposure participants, 33.33% were permanent workers at the plant, compared with 17.28% from the low exposure group. Although between-group differences in socioeconomic markers, such as income, education, and occupation, were not statistically significant, there was a slight trend of higher exposure parents having higher socioeconomic status, with higher average salaries and higher attained education than the low exposure group parents (data not shown). 

### 3.2. Exposure Status 

For the overall external exposure, the high exposure group were with significantly closer distance of an average of 6.33 km from the plant than the low exposure group with an average of 13.16 km from the plant (Table 1). For internal exposure levels, it showed that significant statistical differences were found between urinary levels of chromium, manganese, nickel, copper, arsenic, strontium, cadmium, and lead, with all but copper higher in the high exposure group after adjusting for household smoking, parental work history at the petrochemical plant, age, and gender. The urinary vanadium, mercury, and thallium levels were not statistically significant between high and low exposure groups (Table 1).

### 3.3. Oxidative Stress Status 

Table 1 shows the adjusted means of urinary oxidative stress biomarkers, with the covariates of total dietary antioxidant intake per week, gender, household smoking, age, and parental work history at the petrochemical plant. After adjusting for covariates, none of the differences in means of the high and low exposure groups remained statistically significant. Among these four oxidative stress markers, only 8-OHdG showed slight differences between the high and low exposure groups before adjusting for confounders, with means of 8.53 μg/g-creatinine and 7.18 μg/g-creatinine, respectively, but this difference became insignificant after adjusting for confounders. 

### 3.4. Antioxidant Intake and Nutritional Patterns 

Table 2 showed the comparison of total antioxidant intake and the top five antioxidant ingestion food between high and low exposure groups. Total antioxidant intake per week varied between the exposure groups, with means of 43.18 and 34.20 mmol/week for the high and low exposure groups, respectively. However, this difference was not statistically significant. Additionally, the dietary amount of the highest antioxidant intake foods was similar between the two groups with no statistically significant differences, with the exception of intake of berries per week. 

For all study subjects, among all 45 food groups, not including vitamins or oils, berries were the highest contributor to total antioxidant intake per week (6.10 mmol/week). Sugar tea, dark chocolate, guava, and sugarless tea were the next four most significant contributors to overall antioxidant intake, with contributions of 4.28 mmol/week, 3.09 mmol/week, 3.04 mmol/week, and 2.38 mmol/week, respectively (data not shown).

### 3.5. Effect of Antioxidant Intake on the Association between Metal Exposure and Oxidative Stress

Table 3 presented the associations between each heavy metal and each oxidative stress biomarker for all participants pooled together coupled with the coefficient of antioxidant intake. It showed the obvious associations between metal exposure and oxidative stress. As 8-OHdG was the primary oxidative stress biomarker in this study, it noted that 8-OHdG had significant associations with 7 metals, compared to 10, 3, and 1 for 4-HNE-MA, 8-isoPF2α, and 8-NO2Gua, respectively. Urinary concentrations of vanadium, chromium, copper, arsenic, strontium, cadmium, and mercury were all significantly associated with an increase in urinary 8-OHdG (*p* < 0.05). A one percent increase in vanadium, chromium, copper, arsenic, strontium, cadmium, and mercury resulted in a marginal increase in 8-OHdG of 0.33%, 0.19%, 0.40%, 0.19%, 0.32%, 0.24%, and 0.37%, respectively. On the other hand, we found that there were no significant associations between antioxidant intake and any of the oxidative stress biomarker levels in the study subjects. 

Table 4 and Table 5 present the associations between each heavy metal and each oxidative stress biomarker for the high exposure group and the low exposure group coupled with the coefficient of antioxidant intake. In the high exposure group, it was noted that 8-OHdG had significant associations with six metals, compared to seven, three, and two for 4-HNE-MA, 8-isoPF_2α_, and 8-NO_2_Gua, respectively. In the low exposure group, it was noted that 8-OHdG had significant associations with one metal, compared to five, one, and two for 4-HNE-MA, 8-isoPF_2α_, and 8-NO_2_Gua, respectively. On the other hand, we found that there was no significant association between antioxidant intake and any of the oxidative stress biomarker levels in these two groups, separately, except only one antioxidant intake protection observed in the association between 4HNE-MA and arsenic exposure in the low exposure group.

### 3.6. The Contributions of Multiple Metal Exposure on Oxidative Stress

**Figure 2:**
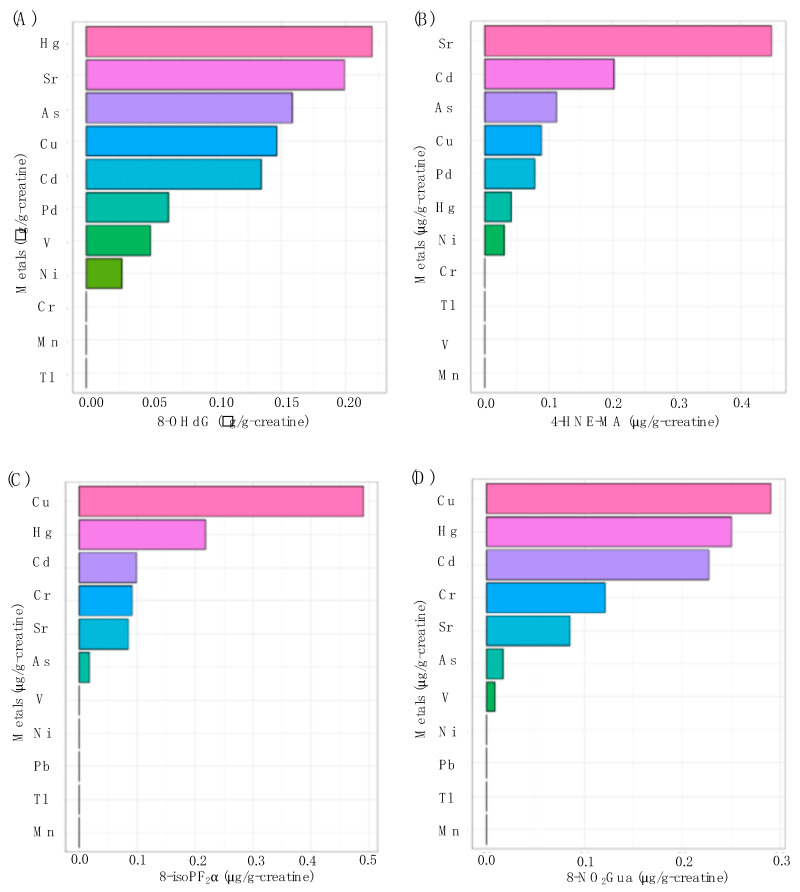
The associations between urinary metal levels and (**A**) 8-OHdG (*p* < 0.01), (**B**) 4-HNE-MA (*p* < 0.01), (**C**) 8-isoPF_2α_ (*p* < 0.01), and (**D**) 8-NO_2_Gua (*p* < 0.01) levels based on weighted quantile sum (WQS) regression analysis.

Figure 2 shows the contributions of multiple urinary metal levels on each urinary oxidative stress biomarker levels individually by WQS regression model. As shown in Figure 2A, the highest contributors to the oxidative stress biomarker of 8-OHdG were Hg, Sr, As, Cu, and Cd, making up 22.0, 19.9, 15.9, 14.7, and 13.5 percent of the total contribution, respectively. Figure 2B shows that Sr was the highest contributor to 4-HNE-MA at 44.7%. The highest contributor to 8-isoPF_2α_ was Cu for 49.1% in Figure 2C. Additionally, V, Hg, and Sr were the highest contributors to 8-NO_2_Gua at 29.1, 25.0, and 22.7 percent, respectively (Figure 2D). All four WQS regression models were statistically significant and showed positive associations between the heavy metals and the oxidative stress biomarker outcomes.

## 4. Discussion

For the petrochemical-related metal exposure, the present study indicated that the study subjects lived in the areas nearby were with significantly higher urinary metal levels except only for Cu with the reverse result (Table 1), and there was limited research that revealed the internal metal exposure dose in young children like the present study. In addition, our previous studies investigated the obviously elevated urinary metal levels in all of the residents with different age groups, including elders, adults, and teenagers [7,33]. We further conducted the distance-to-source analysis, and it revealed that the increased concentrations of most urinary heavy metals in study subjects were associated with the decreased distance from the plant, with the only exception of Cu (data not shown). Among these metals, the Cr, Mn, Ni, and As showed large different exposure levels to possible emission pollution by this petrochemical complex because these metals all were suggested to be the key pollutants by petrochemical industry previously [40,41]. Moreover, the wind direction might influence the metal exposure for our study subjects. Meteorological data was collected from the Central Weather Bureau in Taiwan, and it showed that the kindergartens in our study are located at the east to southeast of the petrochemical complex, and the west to northwest wind direction (downwind for these kindergartens) accounted for 14.3% of all-measured wind directions at the nearest meteorological station. Therefore, the urinary metal levels of residents might be underestimated in the present study, and it should be paid more attention in the downwind season.

On the other hand, Cu was the only nonsignificant finding in this study, and it also indicated the accuracy of the metal exposure representative for the study subjects in these areas because of the main source of Cu exposure are from natural sources (decaying vegetation, forest fires, and sea spray) and anthropogenic emission sources (nonferrous metal production, wood production, iron and steel production) not from the petrochemical industry [42,43]. The previous study indicated that the low exposure area was highly affected by the emission source from vehicles and the nearby urban area in addition to industrial land. Studies also pointed out that the dust from the bare bed of nearby river and agricultural activities might be the source of cooper exposure in the low exposure area [44,45]. Additionally, the previous study, which conducted near a petrochemical industry at the north Kaohsiung, Taiwan, indicated that the PM_2.5_ and PM_10_ levels of copper in the petrochemical area were not significantly different from the nonpetrochemical area [46]. According the above-mentioned, we might clarify the higher urinary copper levels in the low exposure group when compared to those in high exposure group. In the past, the collected air samples in high exposure areas have found that the contents of many metals in PM_10_ were higher during the downwind season to provide the external metal exposure from the petrochemical complex [32]. Nevertheless, the findings in the present study implied the emission-related metal exposure existed even in the children at kindergarten age, and more attention should be paid to the potential adverse health effects of children in this polluted area in the future.

In a previous study conducted in this study area, it was found that teenagers and elders who lived in the high exposure areas were with significantly higher levels of urinary oxidative stress markers [7]. However, the present study did not show any obvious differences in the oxidative stress marker levels of young children between high and low exposure groups (Table 1). One possible reason is due to the different exposure definitions of these two studies. The participants of the previous study were selected from the extreme highest and lowest exposure status with about a two-fold difference in most of the urinary metal levels, but the participants in high and low exposure in the present study were only with relatively slight differences in urinary metal levels. Nevertheless, it showed the significant associations between urinary levels of metals and oxidative stress markers for all subjects in this study (Table 3). When we divided the subjects as the high and low exposure groups, it still showed the significant associations between urinary metal levels and oxidative stress biomarker levels (Table 4 and Table 5). Among these four oxidative stress markers, we found 8-OHdG and 4-HNE-MA were more sensitive to the metal exposure, and the past studies indicated the consistent findings for the application of these two markers in the prediction of the oxidative stress caused by metal exposure [7,47]. In addition, the levels of these two markers in young children in the present study, even with lower urinary metal levels, were obviously higher than those in the teenagers and elders in the past study [7]. Additionally, previous studies supported that the inverse age–oxidative stress relationship could due to the naturally low glutathione levels in children, which means the ability to detoxify reactive oxygen is limited making the younger children more susceptible to oxidative stress [48]. On the other hand, the Hg and Sr showed the dominated contributions for most of the oxidative stress markers in this study (Figure 2). These two metals were the main petrochemical-related emission pollutants [40,41], and several studies have confirmed their effects on the increasing of oxidative stress [49,50,51]. According to the above-mentioned results, young children might be with relatively higher oxidative stress when exposure to metal emission from the petrochemical industry, especially for Hg and Sr, and the subsequent effects of oxidative stress in these young children require further research to clarify.

The differences in dietary antioxidant intake varied slightly between the two groups in the present study, although the high exposure group had a higher average total antioxidant intake (Table 1). Currently, only a few studies have suggested dietary recommendations for vitamin C (the best-known antioxidant), but it has not been clearly defined and it was limited for adults [52,53]. In fact, there was no well-established dietary recommendation for intake of antioxidants per week, especially for children. Limited studies have measured the intake of antioxidants in children populations. One Swedish study analyzed the associations between antioxidant intake and allergic disease on 8-year-old children by applying a food-frequency questionnaire. The result found that the intake of antioxidant, like β-carotene and magnesium in food, had an inverse association with allergic disease, such as rhinitis, atopic sensitization, and asthma [54]. However, most of the previous studies just estimated the single antioxidant not for the total intake of antioxidants in food. On the other hand, there was recently no clear definition on the amount of antioxidant intake enough to achieve the obvious antioxidant effects because of the difficulty to define the level of significant antioxidant protective effect. Therefore, it might be one important reason that we did not observe any significant association between antioxidant intake and oxidative stress even though we divided the subjects as high and low exposure groups) (Table 3, Table 4 and Table 5), even with no contribution for the oxidative stress levels when conducting the WQS model including the antioxidant intake levels (data not shown). 

Previous research has shown that foods with primarily higher antioxidant intake include fruits and vegetables (particularly strawberries, citrus, and kiwi), soybeans, nuts, spices, herbs, yam, mackerel, and so on [38,55]. Among them, soybeans, nuts, strawberries, and kiwi are the more common foods in Taiwan. However, our study subjects ingested the antioxidant mainly from the berries, sugar tea, dark chocolate, guava, and sugarless tea, and this kind of dietary pattern might result in the lower antioxidant intake to against the oxidative stress in the body. In the present study, the average antioxidant intakes were 43.18 and 34.20 mmol/per week for the high and low exposure groups, respectively. Previously, some studies provided the evidence for the Taiwanese with lower antioxidant when compared to other countries. One of the studies found that the vitamin E status in all ages was relatively low in Taiwan when compared to Hungary, Eastern France, and Italy. Another study showed that the serum α-tocopherol (one of the vitamin E) status in Taiwanese children (less than 6 years) was lower than that in France [56]. Meanwhile, the cooking methods might affect the antioxidant properties of food. Previous study showed that water-cooking treatments is a better way to preserved the antioxidant for vegetables when compared to the steaming and frying [57]. In addition, using the microwave to cook vegetables was reported the worst method to retain antioxidants compared to boiling and frying [58]. Unfortunately, these cooking methods described above are common in Taiwanese. For this reason, how to change the dietary habits and cooking way to increase antioxidant intake is an important issue in the future, especially for children living in areas more susceptible to environmental pollution.

Nuclear-factor-erythroid 2-related factor 2 (Nrf2) is considered one kind of redox sensitive transcription factor, and it can regulate expression of numerous detoxifying and antioxidant genes to prevent the oxidative damage [59]. In addition, the metal exposure stimulated the generation of free radicals in vivo, which are often accompanied by activation of transcription factors such as Nrf2. Previous studies found that the level of reactive oxygen species (ROS) significantly increased in Nrf2 knock-down cells under cadmium exposure, which represented Nrf2, played an important role in suppressing the oxidative stress induced by cadmium exposure [60]. Studies have indicated that the consumption of antioxidants have influence on modulation of Nrf2. For instance, the previous study pointed out that the Epigallocatechin-3-gallate, a major polyphenol in green tea, has been shown to induce expression of Nrf2 [61]. According to the above studies, the antioxidants might, through the modulation of Nrf2, lower the oxidative damage. However, the consumption of antioxidants in our study was not enough to restrain the metal-induced oxidative stress.

This study provided the first look at young children’s metal exposure associated with the oxidative stress near the petrochemical industry in Central Taiwan as well as their antioxidant dietary patterns. Traditional studies of exposure assessment usually conducted in combination with a greater view of the paths of exposure and possible adverse effects. Nevertheless, the present study based on the framework of simultaneous assessment of both positive and negative aspects of health effect to provide further insight into the exposure risk factors in this population of children. However, there were several limitations in the current study. First, this cross-sectional study might be difficult to represent the long-term exposure situation to observe the health effects for these young children. Nevertheless, the petrochemical complex in our study started to operate at 1999 for more than 20 years, and it could be considered that the emitted pollutants from the complex were continuous and significant. In addition, the rural socioeconomic status of this study area resulted in most of the kindergarten children who grew up locally. Therefore, it can be expected that these young children were affected by a long-term exposure, and it should not have the directional bias on the exposure results in the present study. 

Second, this study did not consider other sources of potential oxidative stress besides heavy metal exposure. The polycyclic aromatic hydrocarbons and volatile organic compounds emitted from the petrochemical industry were considered to cause the oxidative stress [62]. The heavy metal pollutants were representative of those pollutants from the petrochemical industry in the present study, but it is necessary to clarify the oxidative effects of other exposure source on the young children in advanced studies. Third, it might be considered that the diet of young children is changing and unstable. However, the children in our study are mostly over 4 years old and their chewing and swallowing ability is relatively developed and can have a diet similar to that of adults [63]. Meanwhile, these children were all in kindergartens that provide regular meals to the students, so the dietary pattern of the children in this study is relatively stable for a long time. Fourth, this study did not consider the other source of antioxidant except for food, and the nutritional supplements are one main source of antioxidant for human beings. Usually, the young children would not take those supplements unless they are with some health condition such as specific disease or malnutrition. Therefore, it would be reliable to estimate the mainly antioxidant intake from the dietary of young children in the present study. 

## 5. Conclusions

The World Health Organization estimated that every year 4.2 million premature deaths were attributable to outdoor air pollution. The results of the current study added evidence to environmental health risks presented in previous studies in the study areas, especially for young children. In this study, distance to the industrial exposure site was significantly associated with urinary heavy metal concentrations, and heavy metal concentrations were significantly associated with oxidative stress as measured with 8-OHdG and 4-HNE-MA. Oxidative stress is a harmful mechanism that can cause mortality and morbidity in the form of a myriad of diseases. The results of this study found clear associations between heavy metal exposure and oxidative stress in this area in Central Taiwan. 

Previous studies have indicated that dietary antioxidant intake may provide a protective effect against harmful pollutants. However, the results in the present study cannot provide strong evidence to support this theory. It is possible that the number of dietary antioxidants was not large enough to offset the harmful effects of industrial pollutants in this population. Additionally, low dietary variance in antioxidant intake of the young children in this population may also account for the null association. However, the potential health risk still remains in the study subjects living near a petrochemical complex. Therefore, we suggest the residents, especially for kindergarten children, should lower the chance to expose to heavy metals, and could intake more food rich in antioxidants, such as soybeans, nuts, strawberries, and kiwi, which are common in Taiwan. In addition, the kindergarten in this area could adjust the menu of school lunch to contain more antioxidant-rich food. 

Further studies are needed to determine other factors that may be affecting children’s oxidative stress in this area and should investigate ways to limit exposure outcomes for children, either through stronger dietary precautionary measures, stricter regulation of industry, or both. Given the different roles protective factors play in different oxidative stress outcome measurements, investigating the interplay of industrial pollution, antioxidant intake, and other potential beneficial effects should be explored in advanced.

## Figures and Tables

**Table 1:** Comparison of basic characteristics, urinary heavy metal exposure levels, and urinary oxidative stress biomarker levels between high and low exposure groups.

Variables	Total (n = 168)	High (n = 87)	Low (n = 81)	*p ^a^*
Basic characteristics *^a^*			
Age, mean ± SD (years)	5.42 ± 0.80	5.40 ± 0.84	5.44 ± 0.76	0.73
Male, n(%)	83 (49.4)	50(57.41)	33(40.74)	0.04
Household smoking, n (%)	48 (28.6)	22(25.29)	26(32.10)	0.47
Single parent, n(%)	15 (8.93)	5(5.75)	10(12.35)	0.21
Work history at complex *^b^*, n (%)				0.06
None	75 (44.64)	34(39.08)	41(50.62)	
Current permanent worker	43 (25.59)	29(33.33)	14(17.28)	
Current temporary worker	19 (11.31)	7(8.05)	12(14.81)	
Past permanent or temporary worker	28 (16.67)	17(19.54)	14(17.28)	
External exposures, mean ± SD				
Distance to complex *^c^* (km)	9.82 ± 4.64	6.33 ± 2.82	13.16 ± 1.87	0.00
Internal exposures *^d, e^*, mean ± SD				
Vanadium	0.47 ± 0.23	0.47 ± 0.22	0.47 ± 0.23	0.45
Chromium	1.53 ± 1.18	2.02 ± 1.28	0.98 ± 0.74	0.00
Manganese	1.02 ± 1.73	1.06 ± 1.00	0.97 ± 2.30	0.00
Nickel	4.22 ± 3.46	4.77 ± 3.25	3.61 ± 3.62	0.00
Copper	17.93 ± 7.23	15.86 ± 6.44	20.29 ± 7.39	0.00
Arsenic	94.89 ± 105.45	100.06 ± 59.79	89.04 ± 140.63	0.00
Strontium	200.98 ± 137.89	219.71 ± 137.73	179.78 ± 135.97	0.04
Cadmium	0.085 ± 0.05	0.09 ± 0.04	0.07 ± 0.05	0.00
Mercury	1.85 ± 0.86	1.81 ± 0.79	1.89 ± 0.94	0.77
Thallium	0.21 ± 0.14	0.20 ± 0.13	0.23 ± 0.16	0.40
Lead	1.47 ± 1.12	1.66 ± 1.28	1.24 ± 0.86	0.01
Oxidative stress *^d, e^*, mean ± SD				
8-OHdG	7.90 ± 4.10	8.53 ± 4.69	7.18 ± 3.20	0.14
HNE-MA	47.23 ± 44.80	47.21 ± 47.24	47.24 ± 42.22	0.76
8-isoPF_2α_	11.14 ± 9.84	9.63 ± 5.02	12.85 ± 13.19	0.26
8-NO_2_Gua	0.39 ± 0.41	0.35 ± 0.35	0.44 ± 0.47	0.81

*^a^* Comparison of basic characteristics between the high and low exposure groups for continuous variables by Student’s t-test, and for discrete variables by Chi-squared test. Urinary exposure and oxidative stress biomarker concentrations are log-transformed, high and low exposure groups compared by ANCOVA test adjusting gender, household smoking, age, and parental work history at the petrochemical plant. *^b^* Work history at complex: Parental work history at complex. *^c^* Distance to complex: Average of home-to-complex and kindergarten-to-complex distance, estimating 8 h at kindergarten and 16 h at home per day. *^d^* Unit: µg/g-creatinine. *^e^* High exposure group, n = 76; low exposure group, n = 68.

**Table 2:** The comparison of total antioxidant intake and dietary pattern between high and low exposure groups.

Variables	High (n = 87)	Low (n = 81)	*p ^a^*
Total antioxidant intake, mean ± SD	43.18 ± 40.32	34.20 ± 21.14	0.07
Foods with highest average antioxidant intake			
Berries	8.08	3.98	0.04
Sugar tea	4.81	3.70	0.24
Dark chocolate	3.64	2.50	0.10
Guava	3.02	3.06	0.95
Sugarless tea	2.79	1.95	0.41

*^a^* Comparison of antioxidant intake in foods between the high and low exposure groups was performed using Student’s t-test. Unit: mmol/week.

**Table 3:** The associations between heavy metal exposure and oxidative stress with the antioxidant intake for study subjects (n = 144).

	*Stress Marker*	*8−OHdG*				*4HNE−MA*				*8−isoPF_2α_*				*8−NO_2_Gua*			
*Metal*		Metal Est. (CI) ^a^	*p*	AI Est. (CI) ^b^	*p*	Metal Est. (CI) ^a^	*p*	AI Est. (CI) ^b^	*p*	Metal Est. (CI) ^a^	*p*	AI Est. (CI) ^b^	*p*	Metal Est. (CI) ^a^	*p*	AI Est. (CI) ^b^	*p*
Vanadium		0.33 (0.13, 0.53)	<0.01	−0.05 (−0.20, 0.10)	0.52	0.52 (0.26, 0.78)	<0.01	−0.08 (−0.47, 0.12)	0.45	0.17 (−0.03, 0.37)	0.10	0.04 (−0.11, 0.19)	0.57	0.35 (0.05, 0.65)	0.02	0.15 (−0.08, 0.37)	0.21
Chromium		0.19 (0.01, 0.05)	<0.01	−0.05 (−0.20, 0.10)	0.54	0.42 (0.25, 0.59)	<0.01	−0.08 (−0.27, 0.12)	0.43	0.04 (−0.10, 0.18)	0.57	0.04 (−0.11, 0.20)	0.57	0.10 (−0.11, 0.30)	0.36	0.15 (−0.08, 0.38)	0.21
Manganese		−0.00 (−0.10, 0.10)	1.00	−0.05 (−0.20, 0.11)	0.56	0.03 (−0.12, 0.16)	0.70	−0.07 (−0.28, 0.14)	0.51	−0.09 (−0.19, 0.00)	0.08	0.04 (−0.12, 0.19)	0.64	0.12 (−0.03, 0.27)	0.12	0.16 (−0.07, 0.39)	0.17
Nickel		0.12 (−0.01, 0.25)	0.07	−0.03 (−0.19, 0.12)	0.67	0.19 (0.02, 0.37)	0.03	−0.05 (−0.26, 0.15)	0.61	0.02 (−0.11, 0.16)	0.72	0.05 (−0.11, 0.20)	0.55	0.09 (−0.11, 0.28)	0.40	0.16 (−0.08, 0.39)	0.19
Copper		0.40 (0.14, 0.66)	<0.01	−0.05 (−0.20, 0.37)	0.55	0.47 (0.12, 0.82)	<0.01	−0.07 (−0.28, 0.13)	0.48	0.51 (0.26, 0.76)	<0.01	0.04 (−0.10, 0.19)	0.54	0.16 (−0.23, 0.56)	0.42	0.15 (−0.08, 0.38)	0.21
Arsenic		0.19 (0.03, 0.34)	0.02	−0.07 (−0.23, 0.08)	0.36	0.41 (0.21, 0.61)	<0.01	−0.13 (−0.33, 0.07)	0.20	0.15 (−0.00, 0.31)	0.05	0.02 (−0.13, 0.18)	0.75	0.21 (−0.02, 0.45)	0.07	0.12 (−0.11, 0.35)	0.31
Strontium		0.32 (0.17, 0.48)	<0.01	−0.05 (−0.20, 0.10)	0.50	0.59 (0.40, 0.78)	<0.01	−0.08 (−0.26, 0.10)	0.39	0.18 (−0.02, 0.34)	0.02	0.04 (−0.11, 0.19)	0.58	0.22 (−0.02, 0.46)	0.07	0.14 (−0.08, 0.37)	0.21
Cadmium		0.24 (0.07, 0.40)	<0.01	−0.04 (−0.19, 0.11)	0.58	0.36 (0.14, 0.58)	<0.01	−0.07 (−0.27, 0.13)	0.51	0.19 (0.03, 0.36)	0.02	0.05 (−0.10, 0.20)	0.53	0.03 (−0.23, 0.28)	0.83	0.15 (−0.08, 0.38)	0.21
Mercury		0.37 (0.13, 0.60)	<0.01	−0.04 (−0.19, 0.11)	0.59	0.46 (0.15, 0.77)	<0.01	−0.07 (−.27, 0.14)	0.52	0.17 (−0.07, 0.41)	0.15	0.15 (−0.10, 0.20)	0.53	0.35 (−0.00, 0.71)	0.05	0.15 (−0.08, 0.38)	0.19
Thallium		0.14 (−0.03, 0.31)	0.12	−0.05 (−0.20, 0.11)	0.53	0.25 (0.02, 0.48)	0.03	−0.08 (−0.28, 0.13)	0.46	0.04 (−0.13, 0.21)	0.62	0.04 (−0.11, 0.20)	0.57	0.15 (−0.12, 0.41)	0.26	0.14 (−0.09, 0.37)	0.22
Lead		0.12 (−0.01, 0.26)	0.08	−0.06 (−0.21, 0.10)	0.46	0.23 (0.05, 0.41)	0.01	−0.10 (−0.30, 0.11)	0.36	0.08 (−0.06, 0.22)	0.25	0.04 (−0.12, 0.19)	0.63	−0.10 (−0.31, 0.11)	0.34	0.16 (−0.07, 0.39)	0.18

Note: Multiple linear regression model adjusted for age, gender, household smoking, parental work history at complex, and total antioxidant intake. Each metal was tested individually with each individual oxidative stress biomarker. All estimates of metals, oxidative stress biomarkers, and antioxidant intake were logged. ^a^ Metal estimate (95% confidence interval), ^b^ Antioxidant intake (AI) estimate (95% confidence interval).

**Table 4:** The associations between heavy metal exposure and oxidative stress with the antioxidant intake for study subjects in high exposure group (n = 76).

	*Stress Marker*	*8−OHdG*		*4HNE−MA*		*8−isoPF_2α_*		*8−NO_2_Gua*	
*Metal*		Metal Est. (CI) ^a^	AI Est. (CI) ^b^	Metal Est. (CI) ^a^	AI Est. (CI) ^b^	Metal Est. (CI) ^a^	AI Est. (CI) ^b^	Metal Est. (CI) ^a^	AI Est. (CI) ^b^
Vanadium		0.47 * (0.15, 0.78)	−0.00 (−0.21, 0.21)	0.47 * (0.10, 0.85)	0.05 (−0.20, 0.29)	0.15 (−0.09, 0.39)	0.06 (−0.10, 0.22)	0.35 (−0.04, 0.73)	−0.01 (−0.26, 0.25)
Chromium		0.58 * (0.15, 1.00)	−0.03 (−0.24, 0.18)	0.88 * (0.40, 1.35)	−0.01 (−0.25, 0.23)	0.38 * (0.07, 0.69)	0.03 (−0.13, 0.19)	0.53* (0.02, 1.03)	−0.04 (−0.29, 0.22)
Manganese		−0.13 (−0.31, 0.06)	0.00 (−0.22, 0.22)	−0.07 (−0.29s, 0.14)	0.06 (−0.20, 0.32)	−0.08 (−0.21, 0.06)	0.05 (−0.11, 0.21)	0.16 (−0.06, 0.37)	0.04 (−0.21, 0.30)
Nickel		0.07 (−0.23, 0.37)	0.02 (−0.20, 0.24)	0.22 (−0.13, 0.57)	0.07 (−0.19, 0.33)	0.07 (−0.15, 0.29)	0.06 (−0.10, 0.22)	0.37 * (0.03, 0.72)	0.01 (−0.24, 0.26)
Copper		0.59 * (0.23, 0.95)	0.02 (−0.18, 0.23)	0.54 * (0.11, 0.97)	0.07 (−0.18, 0.32)	0.27 (−0.00, 0.55)	0.06 (−0.09, 0.22)	0.20 (−0.25, 0.65)	0.01 (−0.25, 0.27)
Arsenic		0.30 (−0.03, 0.63)	−0.01 (−0.23, 0.21)	0.55 * (0.18, 0.93)	0.00 (−0.24, 0.25)	0.27 * (0.03, 0.51)	0.03 (−0.13, 0.19)	−0.01 (−0.40, 0.39)	0.01 (−0.25, 0.28)
Strontium		0.49 * (0.23, 0.75)	0.01 (−0.19, 0.21)	0.70 * (0.42, 0.99)	0.06 (−0.17, 0.28)	0.29 * (0.09, 0.48)	0.06 (−0.09, 0.21)	0.21 (−0.11, 0.54)	0.01 (−0.25, 0.26)
Cadmium		0.29 (−0.00, 0.59)	0.01 (−0.21, 0.22)	0.18 (−0.18, 0.54)	0.06 (−0.20, 0.32)	0.15 (−0.07, 0.37)	0.06 (−0.10, 0.22)	0.06 (−0.30, 0.42)	0.01 (−0.25, 0.27)
Mercury		0.49 * (0.12, 0.85)	0.03 (−0.18, 0.24)	0.52 * (0.09, 0.95)	0.08 (−0.17, 0.33)	0.15 (−0.13, 0.43)	0.07 (−0.09, 0.23)	0.18 (−0.27, 0.62)	0.02 (−0.24, 0.27)
Thallium		0.21 (−0.06, 0.48)	0.01 (−0.21, 0.22)	0.24 (−0.08, 0.55)	0.05 (−0.20, 0.31)	0.04 (−0.16, 0.24)	0.06 (−0.10, 0.22)	0.09 (−0.23, 0.41)	0.01 (−0.25, 0.27)
Lead		0.26 * (0.05, 0.46)	0.02 (−0.20, 0.23)in table footer.	0.30 * (0.06, 0.53)	0.06 (−0.18, 0.31)	0.14 (−0.00, 0.30)	0.06 (−0.10, 0.22)	−0.02 (−0.27, 0.23)	0.01 (−0.25, 0.18)

Note: Multiple linear regression model adjusted for age, gender, household smoking, parental work history at complex, and total antioxidant intake. Each metal was tested individually with each individual oxidative stress biomarker. All estimates of metals, oxidative stress biomarkers, and antioxidant intake were logged. ^a^ Metal estimate (95% confidence interval). ^b^ Antioxidant intake (AI) estimate (95% confidence interval), * *p* < 0.05.

**Table 5:** The associations between heavy metal exposure and oxidative stress with the antioxidant intake for study subjects in low exposure group (n = 68).

	*Stress Marker*	*8−OHdG*		*4HNE−MA*		*8−isoPF_2α_*		*8−NO_2_Gua*	
*Metal*		Metal Est. (CI)	AI Est. (CI) ^b^	Metal Est. (CI)	AI Est. (CI) ^b^	Metal Est. (CI)	AI Est. (CI) ^b^	Metal Est. (CI)	AI Est. (CI) ^b^
Vanadium		0.26* (0.03, 0.49)	−0.15 (−0.35, 0.05)	0.53 * (0.18, 0.89)	−0.26 (−0.57, 0.05)	0.10 (−0.23, 0.43)	−0.00 (−0.28, 0.28)	0.34 (−0.13, 0.81)	0.27 (−0.14, 0.68)
Chromium		0.12 (−0.03, 0.28)	−0.13 (−0.33, 0.07)	0.45 * (0.23, 0.67)	−0.20 (−0.50, 0.09)	0.05 (−0.16, 0.27)	0.01 (−0.28, 0.29)	0.13 (−0.18, 0.44)	0.28 (−0.13, 0.70)
Manganese		0.03 (−0.08, 0.15)	−0.15 (−0.36, 0.06)	0.11 (−0.07, 0.29)	−0.26 (−0.58, 0.07)	−0.08 (−0.23, 0.08)	−0.02 (−0.30, 0.27)	0.15 (−0.07, 0.38)	0.29 (−0.12, 0.70)
Nickel		0.10 (−0.05, 0.24)	−0.12 (−0.33, 0.09)	0.16 (−0.06, 0.39)	−0.23 (−0.56, 0.10)	0.04 (−0.15, 0.24)	0.01 (−0.28, 0.30)	0.04 (−0.24, 0.33)	0.27 (−0.15, 0.70)
Copper		0.36 (−0.10, 0.81)	−0.16 (−0.37, 0.04)	0.50 (−0.22, 1.23)	−0.29 (−0.62, 0.04)	1.06 * (0.49, 1.63)	−0.04 (−0.29, 0.22)	−0.14 (−1.06, 0.78)	0.27 (−0.15, 0.68)
Arsenic		0.11 (−0.06, 0.27)	−0.16 (−0.37, 0.04)	0.38 * (0.13, 0.62)	−0.31 * (−0.62, −0.00)	0.14 (−0.07, 0.37)	−0.02 (−0.30, 0.26)	0.35 * (0.04, 0.67)	0.22 (−0.17, 0.62)
Strontium		0.17 (−0.01, 0.35)	−0.14 (−0.35, 0.06)	0.46 * (0.19, 0.73)	−0.25 (−0.55, 0.05)	0.15 (−0.10, 0.40)	0.01 (−0.28, 0.29)	0.29 (−0.08, 0.65)	0.28 (−0.13, 0.68)
Cadmium		0.14 (−0.06, 0.35)	−0.14 (−0.34, 0.07)	0.52 * (0.22, 0.82)	−0.22 (−0.52, 0.09)	0.25 (−0.02, 0.53)	0.03 (−0.25, 0.30)	−0.06 (−0.47, 0.35)	0.26 (−0.16, 0.67)
Mercury		0.19 (−0.11, 0.50)	−0.14 (−0.35, 0.06)	0.37 (−0.10, 0.85)	−0.26 (−0.58, 0.07)	0.14 (−0.28, 0.56)	0.00 (−0.28, 0.29)	0.67 * (0.08, 1.25)	0.29 (−0.11, 0.69)
Thallium		0.09 (−0.11, 0.30)	−0.15 (−0.36, 0.06)	0.28 (−0.04, 0.60)	−0.26 (−0.58, 0.06)	−0.02 (−0.30, 0.26)	−0.00 (−0.29, 0.28)	0.27 (−0.13, 0.67)	0.28 (−0.13, 0.68)
Lead		−0.12 (−0.30, 0.06)	−0.14 (−0.35, 0.06)	0.14 (−0.15, 0.43)	−0.28 (−0.15, 0.23)	0.06 (−0.19, 0.30)	−0.01 (−0.29, 0.28)	−0.17 (−0.53, 0.19)	0.27 (−0.14, 0.68)

Note: Multiple linear regression model adjusted for age, gender, household smoking, parental work history at complex, and total antioxidant intake. Each metal was tested individually with each individual oxidative stress biomarker. All estimates of metals, oxidative stress biomarkers, and antioxidant intake were logged. ^a^ Metal estimate (95% confidence interval). ^b^ Antioxidant intake (AI) estimate (95% confidence interval), * *p* < 0.05.
